# Knowledge, attitude, and practices toward chronic kidney disease among care providers in Jimma town:cross-sectional study

**DOI:** 10.1186/s12889-020-09192-5

**Published:** 2020-07-09

**Authors:** Amare Desalegn Wolide, Kabaye Kumela, Fantu Kerga, Serkadis Debalke, Meskerem Seboka, Birtukan Edilu, Fanta Gashe, Eshetu Mulisa Bobassa

**Affiliations:** 1 Department of Medical Physiology, Institute of Health Sciences, Jimma University, 378 Jimma, Ethiopia; 2 Department of Pharmacy, Institute of Health Sciences, Jimma University, 378 Jimma, Ethiopia; 3 Department of Medical Microbiology, Institute of Health Sciences, Jimma University, 378 Jimma, Ethiopia; 4 Department of Health Service Management, Institute of Health Sciences, Jimma University, 378 Jimma, Ethiopia; 5 Department of Reproductive Health, Institute of Health Sciences, Jimma University, 378 Jimma, Ethiopia

**Keywords:** Care providers, Chronic kidney disease, Kidney disease

## Abstract

**Background:**

Chronic kidney disease (CKD) is a common and growing health problem that requires adequate Knowledge by health care providers to reduce the progress of the disease. Thus, this study aimed to assess the care provider’s Knowledge, attitude, and practices toward CKD.

**Method:**

A cross-sectional study conducted among 326 care providers at Jimma University Specialized hospital and three medium to higher clinics found in Jimma Town. Collected data entered into Epi-Data version 3.1 and exported to SPSS version 21 for windows for data analysis. Descriptive statistics and generalized linear modal used to analyze the data.

**Result:**

The mean age and service year of the participants were 29.68(±4.877) and 4.28(±4.561), respectively. The overall weighted Knowledge, attitude, and practice score of the study participant were 9.0971(8.77, 9.42), 2.53(2.4, 2.65), 10.14(9.94, 10.33) respectively. Over half of the care providers had the awareness to use eGFR to assess kidney function and patient referral to Nephrologists. Also, many care providers knew the five-stage of CKD and the risk factors of CKD, such as diabetes, long-term alcohol consumption, anemia, and cardiovascular disorders, respectively. Care providers had an understanding of late detection, and referral of CKD would increase kidney disease complications. Besides, 275(84.4%) of them are worried about treatment costs related to CKD. Over half of the care providers, 238(73.0%), believed that the Ethiopian ministry of health gave less attention to the problem. Furthermore, 234(71.8%) are interested in studying more on CKD management. Majority 256(78.5%), very likely or likely refer the patient to senior physician and Nephrologist.

**Conclusion:**

Care providers showed enough Knowledge, a favorable attitude, and practice toward CKD.

## Background

Chronic kidney disease (CKD) is a worldwide epidemic health problem of increasing prevalence and costing an enormous burden on healthcare systems [1–3]. The global increase in CKD needs a well-organized preventive strategy mainly by detecting the risk factors [4–7]. According to the American Heart Association (AHA) statement released in 2013, CKD mentioned as a significant risk factor for coronary disease [8–12]. Earlier-stage CKD can also lead to several complications related to anemia and bone mineral metabolism disorders [13]. Despite these known adverse consequences of CKD, the vast majority of the people remain unaware of the disease [14, 15]. Kidney disease (KD) can be diagnosed with simple laboratory procedures. However, people’s practice toward testing (screening) themselves to know the status of CKD is exceptionally very low.

Further, awareness of CKD remains unacceptably low among care providers [16–20]. Earlier recognition of CKD by nephrologists can slow the progression of the disease [21–25]. However, the late evaluation of CKD patients by nephrologists would increase renal failure [26–29]. A non-nephrologist mostly sees most patients with CKD seeking treatment in tertiary hospitals. The Nephrologist has relatively sufficient awareness related to CKD diagnosis than non-nephrologist [14, 30]. In Ethiopia, because of the poor infrastructure, inadequate laboratory facility, and shortage of human resources in the area, physicians frequently use urine and serum creatinine as a means of CKD diagnosis and its severity. However, serum creatinine may not indeed indicate the different stage of CKD, because creatinine level might increases in the blood after high protein ingestions, intense exercise and after taking some drugs such as Cimetidine, Trimethoprim, Pyrimethamine, Salicylates, Phenacemide, Corticosteroids, and Vitamin D derivatives [31]. Increasing evidence indicates that the CKD burden is growing in developing countries because of increased risk factors such as high blood pressure and diabetes mellitus [32–34]. According to the unpublished study, renal disease covers 1.2–6% of adult hospital medical admissions in Ethiopia. Chronic glomerulonephritis, diabetes, and hypertension are the leading causes of chronic kidney disease in Ethiopia.

Additionally, besides the low number of nephrologists in the country, a deficient level of awareness about kidney diseases, risk factors, diagnosis, and management among care providers is believed to be very low. Thus, this study proposed to see the level of care providers’ Knowledge, attitude, and practices (KAP) toward CKD. Farther more, the investigators believe that the study will bring change in the area by identifying gaps for future direction and better patient approach regarding CKD management.

## Methods

### Study design and study setting

A cross-sectional study was conducted at Jimma University Specialized Hospital (JUSH) and three leading higher private clinics, Jimma town, Ethiopia from February 25, 2018, to June 21, 2018. The University hospital is the only specialized referral hospital in the Southwestern part of Ethiopia, serving approximately 12 million inhabitants.

### Sample size and sampling method

The sample size was determined based on the single population proportion formula using Z2 × p × q/d2 with the assumption of the prevalence of 50% for Knowledge, attitudes, and practices (KAP) of chronic kidney disease, a 95% confidence interval and a margin error of 5%. Finally, adding a 10% non-response rate, the total sample size was 422. Study subjects recruited using a non-probability purposive sampling method.

### Inclusion and exclusion criteria

The study covered a wide variety of care provider professionals found in the internal medicine wards, Pediatrics wards, gynecology and obstetrics ward, surgery ward, and many others. General practitioner, resident doctors, specialist doctors, and other health sciences professionals were eligible for the study. Then, consenting professionals received questions on Knowledge, attitude, and practice on chronic kidney diseases.

### Method of data collection

We developed a questionnaire after an extensive literature review to include items assessing Knowledge, attitudes, and practices (KAP) on CKD. Experts in the area evaluated issues difficultly and discrimination in the survey. We did the pre-test among volunteer health care providers to figure out problems in the questionnaire. The data collectors and supervisors discussed the study thoroughly among themselves before data collection. Cronbach’s alpha (> 0.62) used to examine internal consistency and reliability. The questionnaire divided into two sections with the first section comprising the socio-demographic future, service year, and type of area (specialty) that the health care providers are currently working. The later section contains KAP questions to assess professional KAP of chronic kidney disease. The final version administered as part of this study consisted of 25 items divided into three conceptual domains: Knowledge, attitudes, and practices for kidney disease. We designed the knowledge domain to test the understanding of care providers on the etiologies, diagnosis, and treatment of kidney disease as well as the normal function of the kidneys. Fourteen questions in a four-point response scale (“Yes”, “No,” “Do Not Know,” “Unsure”) prepared to assess Knowledge. Similarly, four questions with a five-point categorical response scale (“Strongly Agree”, “Agree”, “Not sure”, “Disagree”, “Strongly disagree”) prepared to measure care provides attitudes. Finally, the practice domain comprised seven items with a four-point Likert-scale (“Very Unlikely”, “Unlikely”, “Likely”, “Very Likely”) to evaluate care providers’ practice.

### Statistical analysis

Data analysis performed using the International Business Machines Corporation-Statistical Package for Social Sciences program version 21 (IBM-SPSS Statistics 21). Results of the continuous variables expressed as means ± Standard Deviation (SD), whereas frequencies (percentages) used to display the result of categorical variables. The normality of the continuous data viewed by using the Kolmogorov–Smirnov test and the data distribution was normal. Generalized linear model used to analyze Knowledge score and crude associations. A dummy coding of 0 and 1 used to enter the nominal independent variables into the regression model. The possible presence of multicollinearity between independent variables was explored based on the variance inflation factor (VIF). Internal consistency of all types of questions and their subscales assessed using Cronbach’s alpha. All *P* values considered significant at < 0.05. For the knowledge domain, we calculated a composite score (range: 0–14). To do so, we first scored each item as correct (1) or incorrect (0) with the responses ‘Do Not Know’ and ‘Unsure’ treated as incorrect. We then obtained the sum of the 14 scored items. For the attitudes domain, each item scored as “Strongly agree” (1), ‘Agree’ (1) or ‘Not sure’ (0), ‘Disagree’ (0), and ‘Strongly disagree’ (0). For the practices domain, each item scored as ‘Very unlikely’ (1), ‘Unlikely’ (1), ‘Likely’ (0), or ‘Very likely’ (0). The study participants who scored 70% and above in the overall weighted mean score considered as having adequate Knowledge, positive attitude and good practices.

### Ethical considerations

The Ethical Review Board of Jimma University approved the study with the reference number IHRPGD/3019/2019. All ethical requirements stringently ensured to keep participants confidentiality.

## Result

### Participant characteristics

A total of 326 health care professionals involved and the majority of the participants were Male 227(69.6%) and general practitioners 96(29.4%). The mean age and service year of the participants were 29.68(±4.877) and 4.28(±4.561), respectively (Table 1).

**Table 1:** Univariable Linear Regression Models Among Care Providers in Jimma, 2018 (*n* = 326)

Variable	Frequency (%)	Mean (±)	Score B (95% CI)	Mean Score (95%CI)
**Sex**				
Male	227(69.6%)	–	0.875(0.204,1.547)	9.54(9.17,9.91)
Female	99(30.4%)	–	r	8.66(8.11,9.22)
**Age**	–	29.68(±4.877)		
**Service year**	–	4.28(±4.561)		
**Area of specialization**				
General Practitioner	96(29.4%)	–	0.595(− 0.257,1.447)	8.82(8.262,9.363)
Residents Doctors	93(28.5%)	–	1.84(0.979,2.694)*	10.1(9.49,10.65)
Specialist Doctors	68(20.9%)	–	1.72(0.802,2.646)*	9.94(9.3,10.59)
Other Health Sciences^a^	69(21.2%)	–	r	8.22(7.56,8.86)
**Overall weighted Knowledge**				9.0971(8.77,9.42)
**Overall weighted Attitude**				2.53(2.4,2.65)
**Overall weighted Practice**				10.14(9.94,10.33)

Key: *B* Unstandardized Coefficients, *CI* Confidence Interval, *r* Reference Group

* = Significant association (*p* < 0.05)

^a^Other Health Sciences = Nurse, Anesthesia, Clinical Pharmacist and laboratory technologist

### Knowledge

The overall weighted Knowledge, attitude, and practice score of the participant on CKD were 9.0971(8.77, 9.42), 2.53(2.4, 2.65), and 10.14(9.94, 10.33). Male participants had higher mean Knowledge (9.54(9.17, 9.91)) than females. Residents 10.1(9.49, 10.65) had a higher knowledge score than other professionals. Also, male sex 0.875(0.204, 1.547) residents 1.84(0.979, 2.694) and specialist 1.72(0.802, 2.646) showed significant liner associations with the crude knowledge score of chronic kidney disease. The majority of the respondents, 191 (58.6%) knew that eGFR (estimated Glomerular Filtration Rate) is a better way to assess the severity of kidney disease. Also, 180(55.2%) of the participants had a knowledge that eGFR helped in referral to a nephrologist (Q2). The highest proportion of the study participants, 202(62.0%), do not think an age-related reduction of eGFR with no kidney disease leads to low eGFR as far as serum creatinine is in the normal range (Q3). Farther more, the majority of the participants also declared that they did not know about the standard treatment protocol and the Modification of Diet in Renal Disease (MDRD) formula to asses kidney function in patients (Q4). Almost all care providers, 298(91.4%), had the understanding that diabetes and hypertension are possible risk factors of CKD. Furthermore, they knew that long-term alcohol consumption and anemia are the potential risk factors of CKD (Table 2).

**Table 2:** Knowledge on stratified by care providers in Jimma Town, 2018 (*n* = 326)

Knowledge Domain Survey Items		Health Care Professional				Total ***n*** = 326 (100%)
		GP ***n*** = 96 (29.4%)	Resident ***n*** = 93 (28.5%)	Specialist ***n*** = 68 (20.9%)	OHS ***n*** = 69(21.2%)	
Q1. Is eGFR a better way… severity of creatinine alone?	0	41(12.6%)	27(8.3%)	25(7.7%)	42(12.9%)	135(41.4%)
	1	55(16.9%)	66(20.2%)	43(13.2%)	27(8.3%)	**191 (58.6%)**
Q2. Has eGFR helped in referral….. Significantly, elevated?	0	35(10.7%)	51(15.6%)	29(8.9%)	31(9.5%)	146(44.8%)
	1	61(18.7%)	42(12.9%)	39(12.0%)	38(11.7%)	**180(55.2%)**
Q3. Can age related reduction... Urine analysis?	0	55(16.9%)	50(15.3%)	40(12.3%)	57(17.5%)	**202(62.0%)**
	1	41(12.6%)	43(13.2%)	28(8.6%)	12(3.7%)	124(38.0%)
Q4. Are you aware of Modification of diet in Renal Disease formula?	0	36(11.0%)	45(13.8%)	35(10.7%)	14(4.3%)	130(39.9%)
	1	60(18.4%)	48(14.7%)	33(10.1%)	55(16.9%)	**196(60.1%)**
Q5. Kidney problem can be easily detected ….urine color or smell?	0	63(19.3%)	64(19.6%)	37(11.3%)	33(10.1%)	**197(60.4%)**
	1	33(10.1%)	29(8.9%)	31(9.5%)	36(11.0%)	129(39.6%)
Q6. Are you aware the five stages of CKD?	0	39(12.0%)	16(4.9%)	11(3.4%)	45(13.8%)	111(34.0%)
	1	57(17.5%)	77(23.6%)	57(17.5%)	24(7.4%)	**215(66.0%)**
Q7. DM and HBP might cause CKD.	0	11(3.4%)	1(0.3%)	4(1.2%)	12(3.7%)	28(8.6%)
	1	85(26.1%)	92(28.2%)	64(19.6%)	57(17.5%)	**298(91.4%)**
Q8. Long-term Alcohol consumption might cause CKD?	0	39(12.0%)	19(5.8%)	10(3.1%)	9(2.8%)	77(23.6%)
	1	57(17.5%)	74(22.7%)	58(17.8%)	60(18.4%)	**249(76.4%)**
Q9. Anemia and cardiovascular disorders are risky for CKD?	0	11(3.4%)	3(0.9%)	7(2.1%)	20(6.1%)	41(12.6%)
	1	85(26.1%)	90(27.6%)	61(18.7%)	49(15.0%)	**285(87.4%)**
Q10.Early detection of chronic kidney disease saves health care cost	0	20(6.1%)	8(2.5%)	9(2.8%)	9(2.8%)	46(14.1%)
	1	76(23.3%)	85(26.1%)	59(18.1%)	60(18.4%)	**280(85.9%)**
Q11. Late referral to a Nephrologists causes..?	0	19(5.8%)	9(2.8%)	7(2.1%)	22(6.7%)	57(17.5%)
	1	77(23.6%)	84(25.8%)	61(18.7%)	47(14.4%)	**269(82.5%)**
Q12. Do you know any standard treatment guideline of CKD?	0	37(11.3%)	40(12.3%)	35(10.7%)	17(5.2%)	129(39.6%)
	1	59(18.1%)	53(16.3%)	33(10.1%)	52(16.0%)	**197(60.4%)**
Q13. Β-Blockers….and ACEIs?	0	51(15.6%)	40(12.3%)	30(9.2%)	15(4.6%)	136(41.7%)
	1	45(13.8%)	53(16.3%)	38(11.7%)	54(16.6%)	**190(58.3%)**
Q14. Dialysis and organ transplantation?	0	9(2.8%)	3(0.9%)	9(2.8%)	8(2.5%)	29(8.9%)
	1	87(26.7%)	90(27.6%)	59(18.1%)	61(18.7%)	**297(91.1%)**

Key: 0-Incorrect (No, Dont know, Unsure), 1-Correct (Yes)

*GP* General Practitioner, *OHS* Other Health Sciences, *eGFR* estimated Glomerular filtration rate, *CKD* Chronic Kidney Diseases, *DM* Diabetes Mellitus, *ACEI* Angiotensin-converting enzyme (ACE) inhibitors

### Attitude

The majority of the study participants, 275(84.4%), agreed that they worry about treatment cost for CKD (Q1). To the same extent, 219(67.2%) of participants also believed that CKD is a significant public health problem in Ethiopia (Q2), and 238(73.0%) of the participants disagree that the ministry of health is working hard in the prevention of the disease (Q3). Furthermore, 234(71.8%) participants agreed that they need more education on CKD and eGFR (Q4) (Table 3).

**Table 3:** Attitude on Chronic Kidney Disease Stratified by Care Providers in Jimma Town, 2018 (*n* = 326)

Attitude Domain Survey Items		Health Care Professional				Total ***n*** = 326 (100%)
		GP ***n*** = 96 (29.4%)	Resident ***n*** = 93 (28.5%)	Specialist ***n*** = 68 (20.9%)	OHS ***n*** = 69 (21.2%)	
Q1. I often worry about treatment cost for CKD for patients?	0	83(25.5%)	86(26.4%)	49(15.0%)	57(17.5%)	**275(84.4%)**
	1	13(16.9%)	7(20.2%)	19(13.2%)	12(8.3%)	51 (15.6%)
Q2.Kidney disease is a major public health problem in Ethiopia?	0	62(19.0%)	65(19.9%)	39(12.0%)	53(16.3%)	**219(67.2%)**
	1	34(10.4%)	28(8.6%)	29(8.9%)	16(4.9%)	107(32.8%)
Q3.Ethiopian Ministry of Health gives adequate attention?	0	24(7.4%)	20(6.1%)	20(6.1%)	24(7.4%)	88(27.0%)
	1	72(22.1%)	73(22.4%)	48(14.7%)	45(13.8%)	**238(73.0%)**
Q4.Do you need more education on CKD and eGFR?	0	65(19.9%)	66(20.2%)	50(15.3%)	53(16.3%)	**234(71.8%)**
	1	31(9.5%)	27(8.3%)	18(5.5%)	16(4.9%)	92(28.2%)

Key: 0- Agree and strongly agree

1-Unsure, Disagree, strongly disagree

*GP* General Practitioner, *OHS* Other Health Sciences, *eGFR* estimated Glomerular filtration rate, *CKD* Chronic Kidney Diseases, *ACEI* Angiotensin-converting enzyme (ACE) inhibitors

### Practices

The majority of the participants 256(78.5%), Very likely or likely refer patients with CKD to senior physician and Nephrologist (Q1). Similarly, 280(85.9%) and 245(75.2%) of participants Very Unlikely and Unlikely refer patients to get care from a traditional healer (Q2) and treat themselves at home (Q3), respectively. Many participants, 260(79.8%), responded Very Unlikely or Unlikely to prepare weekly/monthly meetings to discuss issues related with CKD (Q7) (Table 4).

**Table 4:** Practice on Chronic Kidney Disease Stratified by Care Providers in Jimma Town, 2018 (*n* = 326)

Practice Domain Survey Items		Health Care Professional				Total ***n*** = 326 (100%)
		GP ***n*** = 96 (29.4%)	Resident ***n*** = 93 (28.5%)	Specialist ***n*** = 68 (20.9%)	OHS ***n*** = 69 (21.2%)	
Q1. How likely would you refer patients to Nephrologists?	0	14(4.3%)	16(4.9%)	28(8.6%)	12(3.7%)	70(21.5%)
	1	82(25.2%)	77(23.6%)	40(12.3%)	57(17.5%)	**256(78.5%)**
Q2. How likely would you..Traditional healer?	0	82(25.2%)	79(24.2%)	63(19.3%)	56(17.2%)	**280(85.9%)**
	1	14(4.3%)	14(4.3%)	5(1.5%)	13(4.0%)	46(14.1%)
Q3. How likely would you …to treat themselves at home?	0	58(17.8%)	78(23.9%)	57(17.5%)	52(16.0%)	**245(75.2%)**
	1	38(11.7%)	15(4.6%)	11(3.4%)	17(5.2%)	81(24.8%)
Q4. How likely you use information from text books or social media?	0	38(11.7%)	45(13.8%)	50(15.3%)	29(8.9%)	162(49.7%)
	1	58(17.8%)	48(14.7%)	18(5.5%)	40(12.3%)	**164(50.3%)**
Q5. Have you ever told your patient preventing kidney disease?	0	24(7.4%)	27(8.3%)	32(9.8%)	14(4.3%)	97(29.8%)
	1	72(22.1%)	66(20.2%)	36(11.0%)	55(16.9%)	**229(70.2%)**
Q6. Does your health facility… regarding kidney disease?	0	74(22.7%)	74(22.7%)	58(17.8%)	54(16.6%)	**260(79.8%)**
	1	22(6.7%)	19(5.8%)	10(3.1%)	15(4.6%)	66(20.2%)
Q7. How likely you use routine urine proteins as diagnosis of CKD?	0	25(7.7%)	25(7.7%)	28(8.6%)	25(7.7%)	103(31.6%)
	1	71(21.8%)	68(20.9%)	40(12.3%)	44(13.5%)	**223(68.4%)**

**Key:**

0. Very Unlikely and Unlikely

1. Very Likely and Likely

*GP* General Practitioner, *OHS* Other Health Sciences, *eGFR* estimated Glomerular filtration rate, *CKD* Chronic Kidney Diseases, *ACEI* Angiotensin-converting enzyme (ACE) inhibitors

## Discussion

This study has assessed the care providers’ Knowledge, attitude, and practices toward chronic kidney disease in Jimma town, Jimma Ethiopia. Overall, study participants involved in this study had enough Knowledge, favorable opinion, and practice toward CKD. Participants knew the essential functions of the kidney, risk factors, and diagnosis of CKD. Renal diseases requires a compressive management of glomerular filtration rate, and need to monitor our blood glucose and blood pressure level quite regularly. The Modification of Diet in Renal Disease (MDRD) is a multicenter clinical trial study designed to assess acceptance, safety, and efficacy of restricted protein and phosphorus diets in patients with progressive renal disease [33]. However, in our study, over half of participants had no the understanding about the MDRD equation. In many developing countries, it is hard to get new and advanced medical information quickly due to a lack of resources to access online and printed guidelines. Besides, no enough training available for care provides to update their Knowledge and skills regularly. A similar report has seen from a Tertiary Care Hospital in Pakistan [34, 35]. Our study participants often prescribe drugs such as βBlockers, Thiazide Diuretics, and Angiotensin Converting Enzyme (ACE) inhibitors to patients with CKD. Rubeen et al. 2009 and many others [32, 36–43] have reported the same finding. Dialysis and kidney transplant are the other treatment options for patients with ESKD. However, accessibility and affordability for dialysis and transplant treatment is a massive problem in Ethiopia. On top of that, getting the right organ and organ donors in Ethiopia is still a problem. Besides, the country has a limited nephrologist who can perform this task [44].

In the current study, care providers showed a keen interest in learning about eGFR and kidney disease. Besides, the majority of the study participants felt that kidney problem in Ethiopia is a public health problem that the minister of health gave less attention to the prevention and treatment of CKD. The reason might be a lack of human resources and infrastructure. Resources is one of the vital element to decrease the spread of diseases and improve health services for patients. Kidney disease is now a health problem that kills a thousand individuals without seeing doctors [45]. To mitigate the problem, the ministry of health needs to make a tremendous effort on the health infrastructure and capacity building in the area. As far as practice parameters are concerned, we identified that the majority of the study participants very likely or likely refer patients to Nephrologists. Furthermore, care providers confirm that they had no regular or periodic meeting to discuss kidney disease. However, they often had a talk with their patients about kidney diseases and preventive measures.

### Limitations

We understand that the current study might suffer from the usual “egg or chicken” dilemma, as the cross-sectional design did not allow for a conclusion about the direction of casualty between the response variable (KAP) and predictor variables. The second limitation of the study may be study subject selection bias and recall bias.

## Conclusion

The overall weighted knowledge score of the participant on CKD was 9.0971(8.77, 9.42), which is above a moderate level. Care providers should update themselves on the current advancement of sciences related to CKD treatment and management. Clinical departments should prepare regular meetings to discuss issues associated with CKD. Furthermore, patient referral to the appropriate care unite should be encouraged. The ministry of health should give adequate attention to the prevention work, as many care providers agreed that CKD is a public health problem in Ethiopia. Thus, health ministry and other concerned bodies should update the status of CKD in the country and should get the attention it deserves on treatment and prevention options. Care providers should also update themselves on the current advancement of sciences related to CKD treatment and management. Clinical departments also should prepare regular meetings to discuss issues associated with CKD.

## Supplementary information

**Additional file 1.** Data collection tool.

## Data Availability

The datasets used and analyzed during the current study available from the corresponding author on reasonable request.

## Abbreviations

ACE: Angiotensin Converting Enzyme, A
HA: American Heart Association
CKD: Chronic Kidney Diseases
eGFR: estimated Glomerular Filtration Rate
ESRD: End Stage Renal Disease
JUSH: Jimma University Specialized Hospital
KAP: Knowledge, Attitude, and Practices
KD: Kidney Diseases
MDRD: Modification of Diet in Renal Disease
SD: Standard Deviation
SPSS: Statistical Package for Social Sciences
VIF: Variance Inflation Factor
