# Supplementary material for: Knowledge, attitude, and practices toward chronic kidney disease among care providers in Jimma town:cross-sectional study
Source: BMC Public Health. 2020 Jul 9;20:1079. doi: 10.1186/s12889-020-09192-5 (PMC7346627; doi:10.1186/s12889-020-09192-5)
Supplement: Supplementary file 1 — Additional file 1. Data collection tool. [file 12889_2020_9192_MOESM1_ESM.docx]

**Data collection tool**

**Identification Code: ­­­­­_____**

**Part I: Socio-demographic Characteristics**

1. Gender:_________
2. Age:________
3. Profession:
   1. Medical Doctor
   2. Resident
   3. Specialist
   4. Other
4. **Service year__________________**

**Part II. Knowledge, Attitude and Practice associated with CKD**

1. **Is eGFR (estimated Glomerular Filtration Rate) a better way of assessing severity of kidney function?**
2. Yes
3. No
4. Don‘t know
5. Not sure
6. **Has eGFR helped in referral to a nephrologist?**
7. Yes
8. No
9. Don‘t know
10. Not sure
11. **Can age related reduction in eGFR without kidney disease lead to low eGFR with normal serum creatinine and normal urine analysis?**
12. Yes
13. No
14. Don‘t know
15. Not sure
16. **Are you aware of Modification of diet in Renal Disease (MDRD) formula is used to asses kidney function in patients with kidney disease?**
17. Yes
18. No
19. Don‘t know
20. Not sure
21. **Kidney problem can be easily detected by observing urine color or smell.**
    1. **Yes**
    2. **No**
    3. **Don’t know**
    4. **Unsure**
22. **Are you aware the five stages of CKD and each stage has its management plan to help reduce complications and help slow down the progression of CKD?**
23. Yes
24. No
25. Don‘t know
26. Not sure
27. **Chronic illness (High blood pressure and DM) might cause CKD**
28. Yes
29. No
30. Don’t know
31. Unsure
32. **Long-term Alcohol consumption might cause CKD.**
    1. Yes
    2. No
    3. Don’t know
    4. Unsure
33. **Anemia and CVD are possible complication of CKD.**
34. Yes
35. No
36. Don’t know
37. Unsure
38. **Early detection of chronic kidney disease saves health care cost**
39. Yes
40. No
41. Don’t know
42. Unsure
43. **Late referral to a Nephrologist causes high morbidity, mortality and rate of hospitalization in CKD patients.**
44. Yes
45. B. No
46. C. Don‘t know
47. D.Notsure
48. **Do you know any standard treatment guideline of CKD management?**
49. Yes
50. No
51. Don‘t know
52. Not sure.
53. **Β-Blockers, Thiazide Diuretics, and ACEIs are some of the drugs have renal protective effect in chronic kidney disease.**
54. Yes
55. No
56. Don’t know
57. Unsure
58. **Dialysis and organ transplantation are options for treatment of ECKD.**
59. Yes
60. No
61. Don’t know
62. Unsure
63. **I often worry about treatment cost for CKD for patients.**
    1. Strongly agrees
    2. Agree
    3. Not sure
    4. Disagree
    5. Strongly disagree
64. **Kidney disease is a major public health problem in Ethiopia.**
    1. Strongly agrees
    2. Agree
    3. Not sure
    4. Disagree
    5. Strongly disagree
65. **Ethiopian Ministry of Health gives adequate attention to kidney disease management and its prevention.**
    1. Strongly agree
    2. Agree
    3. Not sure
    4. Disagree
    5. Strongly disagree
66. **Do you need more education on CKD and eGFR?**
67. Strongly agree
68. Agree
69. Not Sure
70. Disagree
71. strongly disagree
72. **How likely would you be willing to refer patient with CKD to senior physician and Nephrologist?**
73. Very unlikely
74. Unlikely
75. Likely
76. Very likely
77. **How likely would you refer patients to get care from a traditional healer?**
    1. Very unlikely
    2. Unlikely
    3. Likely
    4. Very likely
78. **How likely would you recommend patients with CKD to treat themselves at home?**
79. Very unlikely
80. Unlikely
81. Likely
82. Very likely

1. **How likely would you recommend CKD patient to collect information from textbook, radio, Internet, newspaper, television, and social media?**
2. Very unlikely
3. Unlikely
4. Likely
5. Very likely
6. **Have you ever told your patient about the importance of preventing kidney disease?**
7. Very unlikely
8. Unlikely
9. Likely
10. Very likely
11. **Does your health facility have a weekly/monthly programme focusing on provision of health information regarding kidney disease?**
    1. Very unlikely
    2. Unlikely
    3. Likely
    4. Very likely
12. **Have you ever talked to your patient about the risks for developing CKD?**
    1. Very unlikely
    2. Unlikely
    3. Likely
